# Supplementary material for: Combination of Lactobacillus plantarum HAC03 and Garcinia cambogia Has a Significant Anti-Obesity Effect in Diet-Induced Obesity Mice
Source: Nutrients. 2023 Apr 12;15(8):1859. doi: 10.3390/nu15081859 (PMC10142012; doi:10.3390/nu15081859)
Supplement: Supplementary file 1 [file nutrients-15-01859-s001.zip › nutrients-2332753-supplementary.pdf]

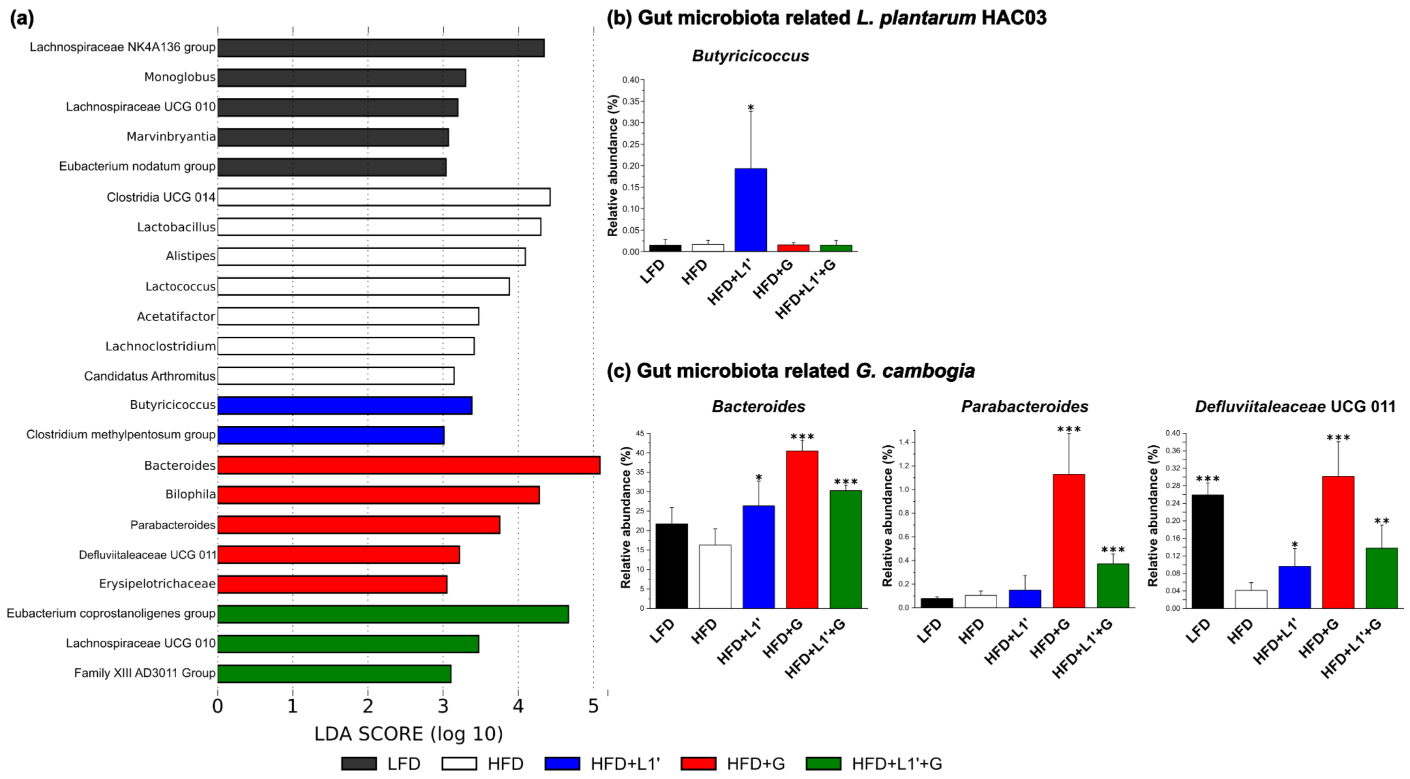

Supplementary Figure S1. Gut microbiota related specific treatment. (a) Linear discriminant analysis (LDA) effect size (LEfSe) with LDA effect size ≥ 2 and alpha ≤ 0.05 for all groups. (b) gut microbiota related *L. plantarum* HAC03 treatment (c) gut microbiota related *G. cambogia* treatment.The data presented as mean ± SD (n = 5). A one-way ANOVA with Dunnett's comparison test was used to compare with HFD groups * $p < 0.05$, ** $p < 0.01$, *** $p < 0.001$.

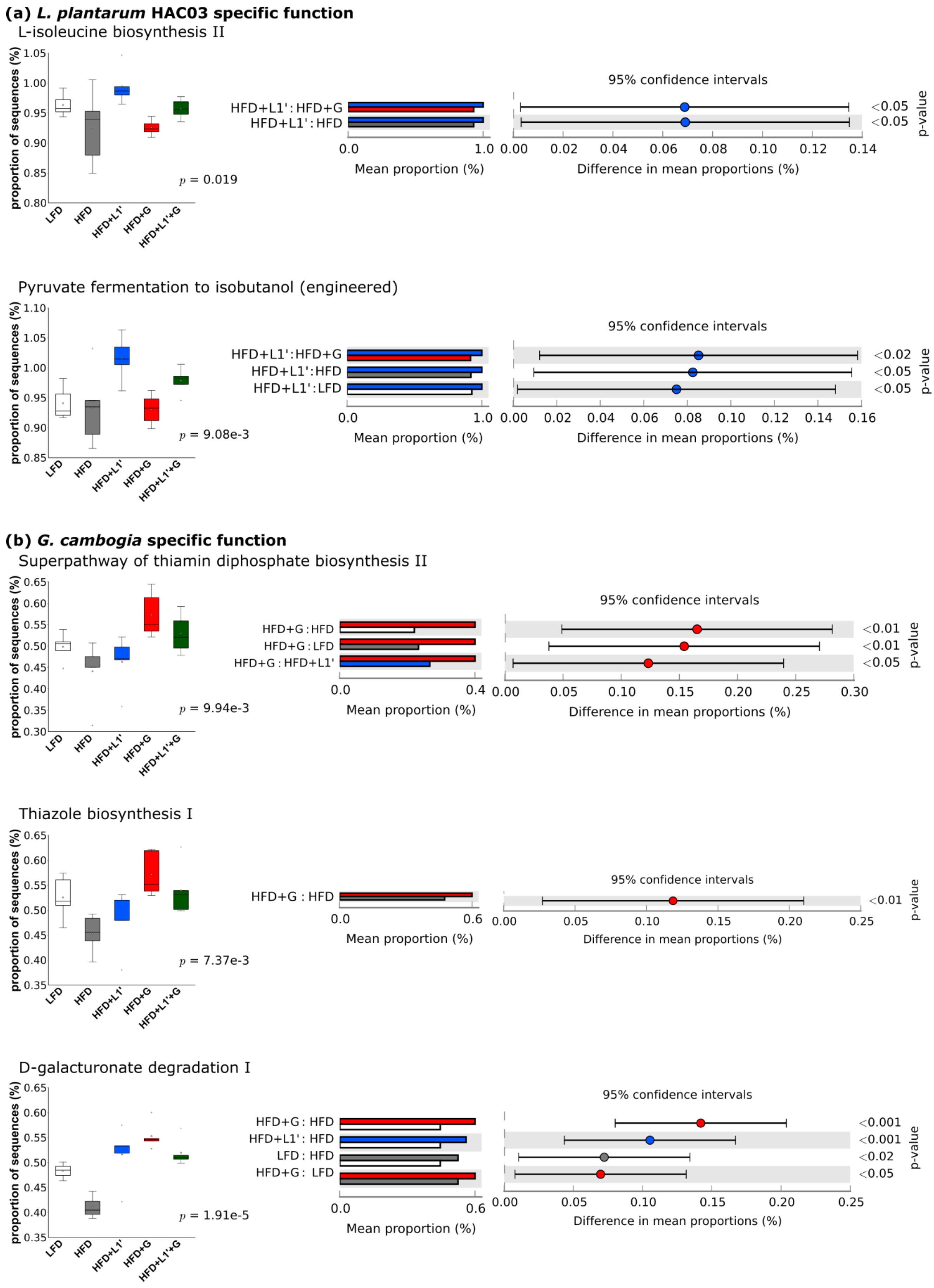

Supplementary Figure S2. Functional potential prediction of specific treatment. (a) functional potential prediction of *L. plantarum* HAC03 treatment. (b) functional potential prediction of *G. cambogia* treatment. The data presented as mean ± SD (n=5). Significance for data is calculated by one-way ANOVA followed by Bonferroni multiple comparison test.
